# Sharp mandibular bone irregularities after lower third molar extraction: Incidence, clinical features and risk factors

**DOI:** 10.4317/medoral.18700

**Published:** 2013-03-25

**Authors:** Daniela Alves-Pereira, Rui Figueiredo, Eduard Valmaseda-Castellón, Daniel M. Laskin, Leonardo Berini-Aytés, Cosme Gay-Escoda

**Affiliations:** 1 DDS. Master degree in Oral Surgery and Implantology. School of Dentistry of the University of Barcelona (Spain); 2 DDS. Associate Professor of Oral Surgery. Professor of the Master degree program in Oral Surgery and Implantology. School of Dentistry of the University of Barcelona (Spain). Researcher of the IDIBELL Institute; 3 DDS, PhD. Professor of Oral Surgery. Professor of the Master degree program in Oral Surgery and Implantology. School of Dentistry of the University of Barcelona (Spain). Researcher of the IDIBELL Institute; 4 DDS, MS, Professor and Chairman Emeritus, Department of Oral and Maxillofacial Surgery, Virginia Commonwealth University, Richmond, Virginia, USA; 5 DDS, MD, PhD. Professor Emeritus of Oral and Maxillofacial Surgery. Professor of the Master degree program in Oral Surgery and Implantology. School of Dentistry of the University of Barcelona (Spain). Researcher of the IDIBELL Institute; 6 DDS, MD, PhD. Chairman and Professor of Oral and Maxillofacial Surgery. Director of the Master degree program in Oral Surgery and Implantology. School of Dentistry of the University of Barcelona. Researcher of the IDIBELL Institute. Oral and Maxillofacial Surgeon of the Teknon Medical Center, Barcelona (Spain)

## Abstract

Objectives: The purpose of this study was to determine the incidence and clinical symptoms associated with sharp mandibular bone irregularities (SMBI) after lower third molar extraction and to identify possible risk factors for this complication. Study Design: A mixed study design was used. A retrospective cohort study of 1432 lower third molar extractions was done to determine the incidence of SMBI and a retrospective case-control study was done to determine potential demographic and etiologic factors by comparing those patients with postoperative SMBI with controls. Results: Twelve SMBI were found (0.84%). Age was the most important risk factor for this complication. The operated side and the presence of an associated radiolucent image were also significantly related to the development of mandibular bone irregularities. The depth of impaction of the tooth might also be an important factor since erupted or nearly erupted third molars were more frequent in the SMBI group. Conclusions: SMBI are a rare postoperative complication after lower third molar removal. Older patients having left side lower third molars removed are more likely to develop this problem. The treatment should be the removal of the irregularity when the patient is symptomatic.

** Key words:**Third molar, postoperative complication, bone irregularities, age.

## Introduction

The most common postoperative complications reported after lower third molar extractions are infection and dry socket (1-4). However, other complications such as loss of periodontal attachment on the adjacent second molar and damage to the inferior alveolar and lingual nerves can also occur (5-7). Less common problems, such as the development of sharp mandibular bone irregularities (SMBI), are usually not mentioned in the literature. This complication consists in the development of a sharp bony margin or fragment located on the lingual aspect of the socket that can cause discomfort to the patient. The objectives of this study were to determine the incidence, the clinical symptoms and the risk factors of the patients that develop SMBI after lower third molar extraction.

## Material and Methods

The initial part of this investigation involved a retrospective analysis of the records of a cohort of 1109 consecutive patients in whom 1432 lower third molars extractions had been performed between July 2005 and February 2007 in the Oral Surgery and Implantology Department of the School of Dentistry of the University of Barcelona (Spain). Patients that developed a postoperative SMBI were identified. A SMBI was defined as the presence of a small, uneven, sharp projection or bony margin located on the lingual aspect of the lower third molar region that developed following the extraction.

In the second part of the investigation, a case-control design was used. Those patients with a SMBI were compared with a random selection of 46 patients without this postoperative complication who had been seen more than one month after the original surgical procedure. The study protocol was approved by the Institutional Review Board (Ethical Committee for Clinical Investigation of the Dental Clinic of the University of Barcelona).

All patients had one lower third molar extracted under sterile conditions at each operation, generally under local anesthesia with 4% Articaine with 1:100.000 epinephrine (Artinibsa; Inibsa, Lliça de Vall, Spain). The technique used was similar to that described in previous reports (1,8). After the operation, an antibiotic (usually amoxicillin 750 mg every 8 hours for 4-7 days [Clamoxyl 750; GlaxoSmithKline, Madrid, Spain]), a non-steroidal anti-inflammatory drug (usually sodium diclofenac 50 mg every 8 hours [Diclofenac Llorens 50 mg; Llorens; Barcelona, Spain] or ibuprofen 600 mg every 8 hours for 4-5 days [Algiasdin 600; Esteve; Barcelona, Spain]), an analgesic (usually metamizol 575 mg every 6 hours for 3-4 days [Nolotil; Boehringer Ingelheim; Sant Cugat del Vallès, Spain]) and a mouthrinse (0.12% chlorhexidine digluconate every 12 hours for 15 days [Chlorhexidina Lacer; Lacer; Barcelona, Spain]) were prescribed. Patients with flapless extractions did not receive antibiotics. Although the systematic use of antibiotics in third molar surgery is controversial, with reports that discourage such prescription (9,10), 2 randomized controlled trials (RCT) published in 2005 (11) and 2007 (12) support such action to prevent infectious and inflammatory complications. Postoperative instructions and use of prescribed drugs were explained to the patients and they were also given a printed handout.

All clinical records were examined by a single investigator (DAP). The following data were retrieved: age, gender, smoking habits, history of preoperative pain or infection, operated side, position of the lower third molar according to the Winter classification, distal space and depth of impaction using the Pell & Gregory classification, degree of soft tissue and bone coverage, presence of a radiolucent lesion associated with the tooth, flap design, need for bone removal and tooth sectioning, and presence of the adjacent lower second molar. Additionally, the following variables were retrieved from the clinical records of patients who developed postoperative SMBI: the time elapsed from extraction of the lower third molar to the diagnosis of a SMBI, the associated clinical symptoms, the treatment and the time from diagnosis to complete healing.

Data were processed with the Statistical Package for the Social Sciences (SPSS version 15.0; SPSS, Chicago, Ill, USA). Pearson’s chi-square, Fisher exact tests and t-student tests were used to compare the groups. The level of significance was set at p<0.05. The data was also explored using binary unconditional logistic regression. The dependent variable was the occurrence of a SMBI and the independent variables were all the remaining preoperative factors. Variables were introduced sequentially with a process based on the change in the likelihood ratio (LR), but the process was completed by entering the variables stepwise. The criterion was to keep all variables that could be associated with the appearance of a SMBI and cause a significant reduction in the -2•log (LR). Assumptions for logistic regression models were checked.

## Results

**Figure 1:**
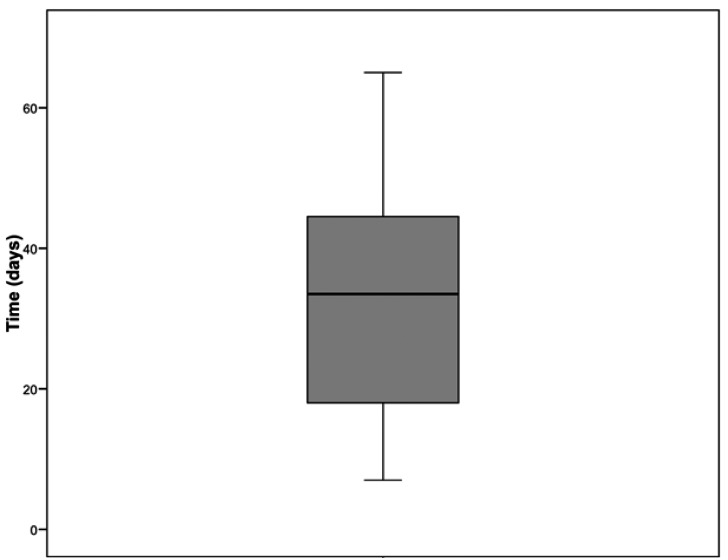
Boxplot representing time elapsed between the diagnosis of the SMBI and complete healing.

A total of 12 SMBI were found on the lingual side of the third molar extraction sockets, which corresponds to an incidence of 0.84% (95% confidence interval (CI): 0% to 2.2%). Nine SMBI were associated with erupted or nearly erupted teeth and 4 with partial bony impactions. The mean age of the patients presenting with this complication was 47.2 years, with a standard deviation (SD) of 14.0 years, while in the control group (CG) the mean age was 28.2 years (SD=10.8 years); this difference was significant (Student t-test for independent samples: t=-5.100; df=56; p= 4.19•10-6). The mean time to diagnosis was 37.3 days (SD=25.2 days). The mean time elapsed between diagnosis and complete healing was 33.2 days (SD=19.9 days) (Fig. 1). The individual data regarding age, gender, time elapsed between tooth extraction and diagnosis and complete healing, symptoms and treatment are shown in  Table 1.

**Table 1:**
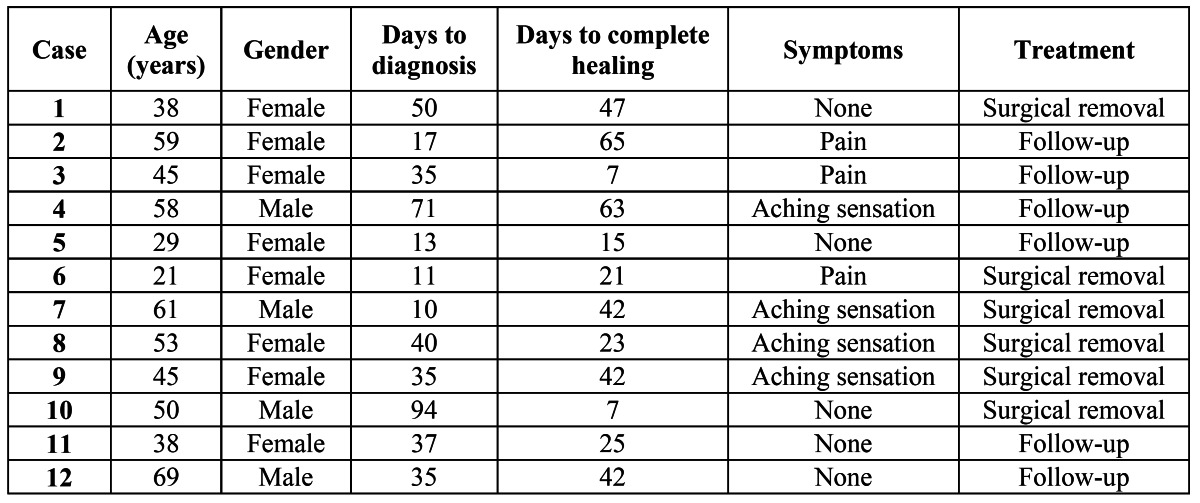
Clinical features of the patients with SMBI after impacted lower third molar extraction.

 Table 2 shows that there was a significant relationship between the occurrence of a SMBI and the side of the impacted third molar (p=0.033) as well as with the presence of a radiolucency (p=0.01). However, the logistic regression model ruled out an association between a radiolucency and development of a SMBI. There was also no significant relationship to gender, smoking habits, flap design, Winter and Pell and Gregory classifications, soft tissue coverage, surgical technique variables (bone removal and tooth sectioning) or the presence of an adjacent second molar (p>0.05) ( Table 2, Table 3).

**Table 2:**
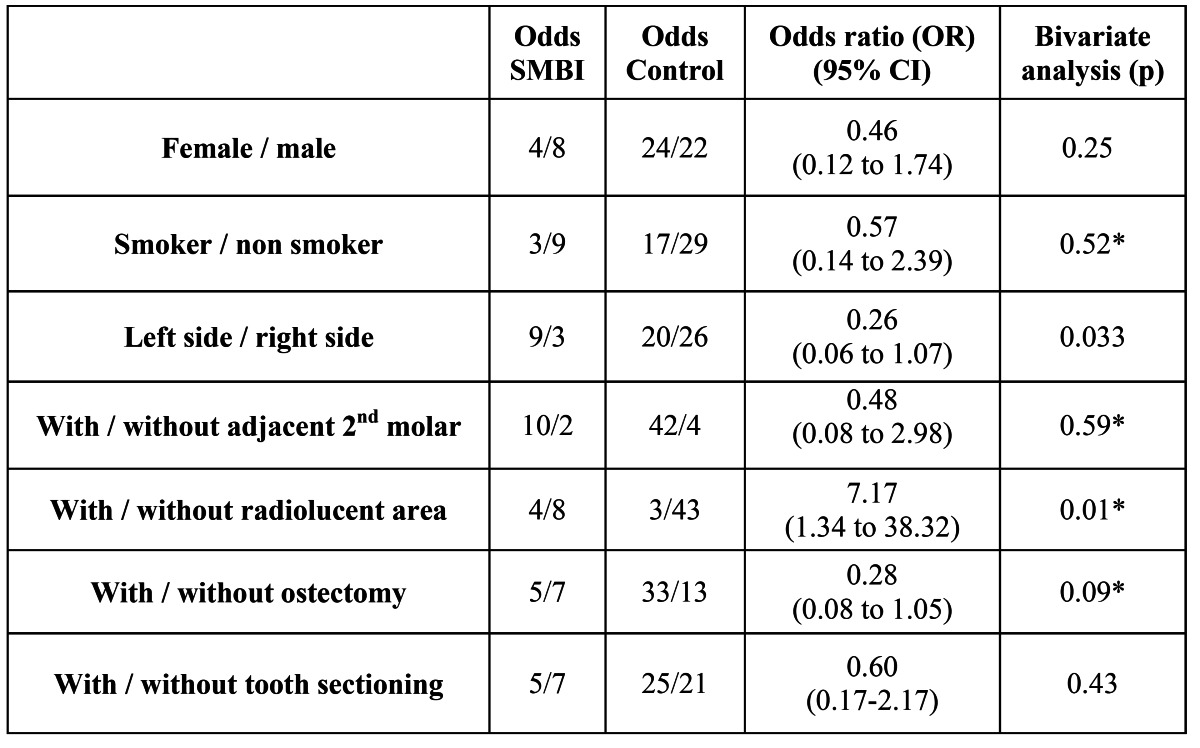
Results of binary variables. Left operated site and radiolucent area showed a statistically significant association to the development of a postoperative SMBI. Significance (p) was calculated with Pearson’s chi-square test except in the cases with *, where a two-tailed Fisher’s exact test was used.

**Table 3:**
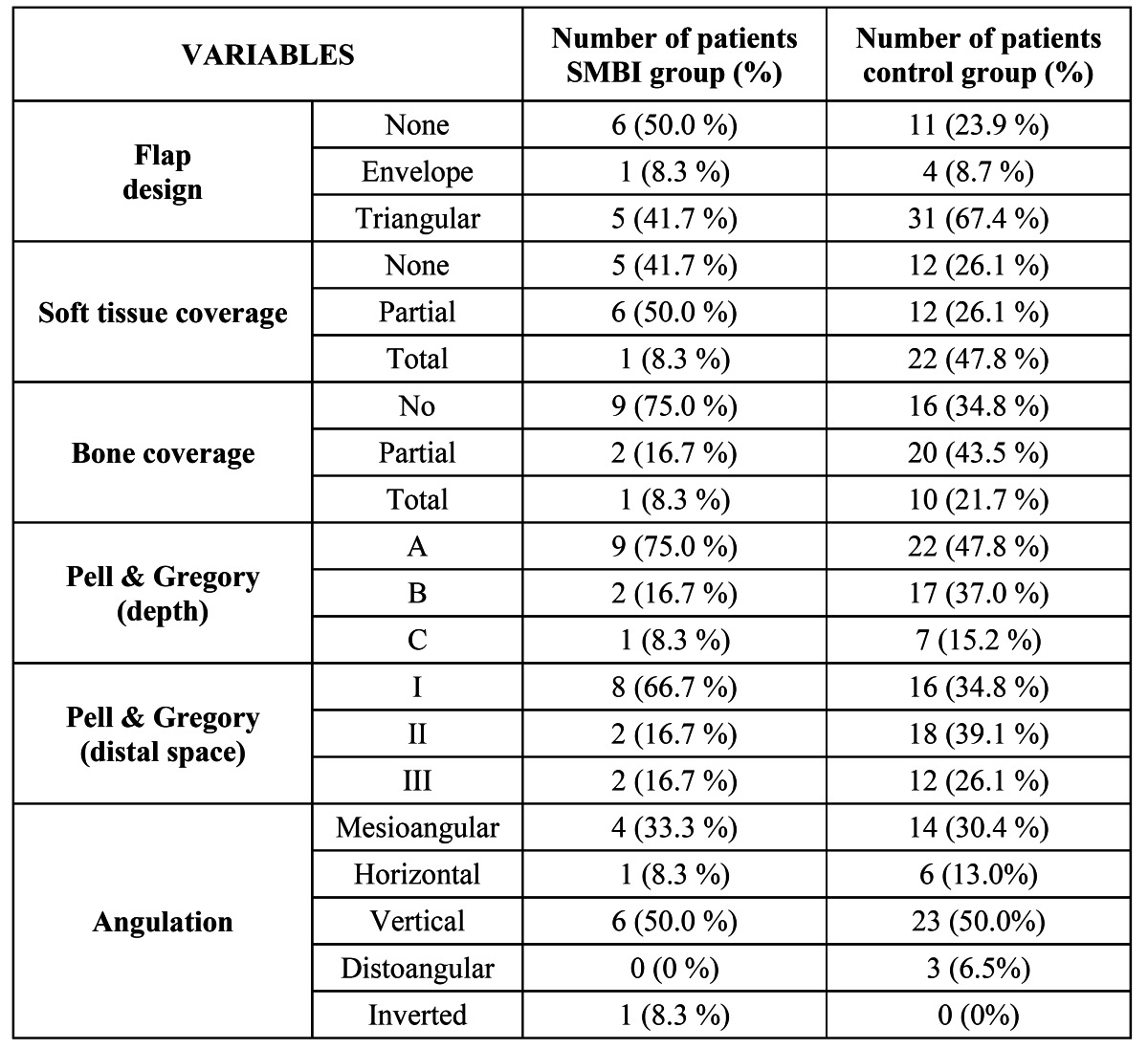
Results of the variables with more than two categories. If the 3 first variables are displayed in 2x2 tables (grouping “envelope flap” with “triangular flap” and “partial retention” with “total retention”) bone coverage would be significantly associated with SMBI (p=0.012).

The logistic regression model for the appearance of a SMBI included only 1 independent variable: patient age (-2•log (LR)=17.293; Nagelkerke’s R2=0.403). The model was significant (Wald=12.30; df=1, p=0.0005), and eβ (the odds ratio for a difference of age of 1 year) was estimated at 1.109 (95 % CI 1.047 to 1.175).

## Discussion

To our knowledge, this is the first study published on the occurrence of SMBI after lower third molar extraction. Such areas are usually painful on palpation and may perforate the overlying mucosa. However, some may remain unnoticed by the patient. The main symptoms related by patients are pain or an annoying sensation in the overlying mucosa. Although the incidence found was rather low, this figure must be interpreted with caution due to the retrospective nature of this study which might have a tendency to underestimate the real incidence, because some patients who develop the problem may not return for care. Despite this relatively low incidence, the clinical symptoms and the delayed recovery justify the need to prevent this complication, because it can have a negative impact on quality of life (13,14). Indeed, although the associated symptoms are usually not severe, they can last for several weeks, until bone remodeling of the sharp irregularity takes place. Sometimes, it is necessary to remove or smooth this irregularity to relieve pain when swallowing or chewing. As can be seen in  Table 1 and fig. 1, the mean time of the symptoms in the affected area is approximately one month.

The etiology of a SMBI after lower third molar extraction can be explained by either a fracture of the cortical plate, especially on the lingual side of the socket which is usually very thin (15), or by the presence of a sharp cortical margin. The fact that most of the cases were erupted or nearly erupted third molars, with extraction involving a simple procedure supports this theory. The buccal-lingual movement used to expand the tooth socket could cause the thin plate to fracture. Other evidence that points toward this mechanism of injury is that older patients seem to be more prone to developing SMBI. This is an important issue, since the reduced bone elasticity in these patients (16), could lead to a greater incidence of bone plate fractures. On the other hand, in partially impacted teeth, most of which had a mesioangular tilt ( Table 3), the location of the crown could result in more inferior positioning and even greater thinning and sharpness of the upper edge of the lingual cortical plate.

We found that extraction of the left lower third molar was associated with a greater incidence of postoperative SMBI than right side removal. Although the dominant hand of the surgeons was not recorded, it is reasonable to assume that most of them were right handed. Therefore, extractions on the left side would be more difficult to visualize and remove and could account for greater bone damage and therefore more SMBI.

Although the bivariate analysis showed a statistically significant association between the presence of a radiolucency and a SMBI, the multivariate analysis failed to show such an association. Although several factors seemed to be involved in the bivariate analysis, only age explained the occurrence of a SMBI, without any additional significant effect of the rest of variables.

The fact that patients may be asymptomatic, or complain of only a slight aching sensation, justifies a conservative approach toward resolving this complication. First, it is essential to determine by palpation whether the cortical plate is loose, because this could cause a foreign body reaction and therefore it should be removed. If the bone is not loose, but causes pain, its removal may still be advisable. In these cases, there are two surgical options. One consists of the trimming of the fragment using the existing perforation of the mucosa, and the other technique requires the raising of a mucoperiosteal flap and elimination of the SMBI using a surgical bur or a bone file. The latter approach is generally preferable because attempting to remove the SMBI through the perforation could result in the creation of a larger dehiscence in the mucosa and the exposure of more cortical bone. SMBI that are asymptomatic should be left alone until spontaneous resolution occurs.

As a conclusion, mandibular sharp bone irregularities after lower third molar extraction are a rare postoperative complication, with an estimated incidence of 0.8%, that could delay the full recovery of patients by several weeks. Older patients with erupted or nearly erupted left lower third molars, are more prone to developing this complication. The treatment should be the removal of the irregularity under local anesthesia when it is symptomatic, and observation for those patients that do not report symptoms.
